# Hyperactive–impulsive ADHD traits predict higher curiosity in adults: evidence from a cross-sectional study

**DOI:** 10.1186/s40359-026-04504-7

**Published:** 2026-04-10

**Authors:** Anne-Laure Le Cunff, Charlotte Russell, Eleanor J. Dommett

**Affiliations:** Institute of Psychiatry, Psychology and Neuroscience, King’s College London, London, UK

**Keywords:** Curiosity, Learning, Exploration, Behavioral traits, ADHD

## Abstract

**Background:**

Attention-deficit/hyperactivity disorder (ADHD) is typically framed in terms of difficulties with sustained attention, behavioral regulation, and impulse control, yet growing work suggests that some associated traits may also relate to potentially adaptive tendencies. Curiosity has been highlighted in qualitative research as one such trait that can function as both a strength and a challenge for adults with ADHD, but quantitative evidence examining its relationship to ADHD traits remains limited. This study examined whether ADHD traits are associated with individual differences in trait curiosity in adults.

**Methods:**

A cross-sectional survey was conducted in 521 adults living in the United Kingdom, of whom 50.7% reported a formal ADHD diagnosis. ADHD traits were assessed using the Adult ADHD Self-Report Scale v1.1 (ASRS-v1.1) and curiosity was measured with the Curiosity and Exploration Inventory-II (CEI-II). Associations between ADHD traits and curiosity were first examined using Pearson correlations. Multiple linear regression analyses were then used to test whether inattention and hyperactivity–impulsivity uniquely predicted curiosity after controlling for age, gender, and education. Finally, curiosity was compared between diagnostic groups using Welch’s *t*-tests, and distribution-based analyses examined co-occurrence of high hyperactivity–impulsivity and high curiosity.

**Results:**

ADHD trait scores showed small-to-moderate positive correlations with overall curiosity and its subdimensions. Participants with ADHD reported moderately higher curiosity than those without a diagnosis. In regression analyses controlling for demographic variables, hyperactivity–impulsivity uniquely predicted curiosity (*β* = 0.26, *p* < .001), whereas inattention did not. This pattern was consistent across diagnostic groups.

**Conclusion:**

Hyperactive–impulsive ADHD traits are associated with higher self-reported curiosity in adults. These findings are consistent with accounts that link impulsivity-related traits to greater engagement with novelty and uncertainty, and suggest that some traits commonly framed as challenges may also relate to tendencies that individuals experience as strengths. Further research is needed to determine whether and when this association translates to functional advantages in educational and occupational settings.

**Supplementary Information:**

The online version contains supplementary material available at 10.1186/s40359-026-04504-7.

## Background

Attention-deficit/hyperactivity disorder (ADHD) is a neurodevelopmental condition affecting approximately 5% of children and adolescents and 3% of adults worldwide [1, 2]. ADHD is traditionally characterized by persistent patterns of inattention and/or hyperactivity–impulsivity that interfere with typical functioning [3]. In research and clinical contexts, these symptoms are often described along two dimensions: inattention, involving difficulties directing or sustaining focus, and hyperactivity–impulsivity, involving restlessness and difficulty inhibiting actions. These dimensions can appear separately or together and are widely used to capture variability in ADHD presentations [4, 5].

However, emerging research calls into question an entirely deficit-focused view of ADHD by examining traits that may also have adaptive aspects [6–10]. In particular, individuals with ADHD have described curiosity as both a strength and a challenge, fueling deep engagement in areas of interest while sometimes leading to difficulty prioritizing tasks and impulsive information-seeking [11, 12]. Curiosity has been described as part of a broader pattern of explorative and novelty-seeking tendencies in ADHD, including being adventurous, trying new experiences, and enjoying learning across diverse topics [7]. Other studies of potential strengths in ADHD place curiosity within a broader construct of ‘cognitive dynamism,’ a cluster of traits also including divergent thinking, hyperfocus, and creativity [10]. Most prior work on this topic has been qualitative [6, 7, 10], though one recent quantitative study examining self-reported strengths found that hyperactivity–impulsivity traits – but not overall ADHD traits – showed a small but significant positive correlation with curiosity [9].

Over a century ago, a possible connection between curiosity and impulsivity was already implied by philosopher and psychologist William James characterising curiosity as “the impulse towards better cognition” [13]. Recent neuroimaging studies support this potential association, with research suggesting that both curiosity and impulsivity rely on similar neural circuits – specifically frontostriatal networks and midbrain dopamine inputs to the striatum and prefrontal cortex – indicating possible shared mechanisms [14]. Building on this work, recent theoretical accounts propose ‘hypercuriosity’ as an impulsive form of exploratory behavior in which heightened responsiveness to stimulation, combined with reduced inhibitory control, leads to rapid engagement with novel or uncertain situations [15]. Although hypercuriosity is not proposed as ADHD-specific, its theorized relationship to impulsivity offers a useful lens to understand how some individuals with ADHD may engage with novelty and uncertainty, situating these behaviors within the broader ADHD phenotype beyond core DSM symptoms.

In addition, established measures of curiosity include dimensions that overlap conceptually with some behaviors seen in ADHD. The 5-Dimensional Curiosity Scale (5DC) identifies Thrill Seeking, which corresponds to the novelty-seeking and exploration behaviors often observed in ADHD, and Deprivation Sensitivity, driven by the need to quickly resolve uncertainty [16]. Similarly, the Curiosity and Exploration Inventory-II (CEI-II) identifies two key dimensions of trait curiosity: motivation to seek out knowledge and new experiences (Stretching), and willingness to embrace the novel, uncertain, and unpredictable nature of everyday life (Embracing) [17]. The Five Factor Model personality trait ‘openness’, which encompasses intellectual curiosity and a preference for variety [18], has also been linked to specific neurocognitive profiles within ADHD. In one study, higher openness was associated with the ADHD subgroup characterized by poorer attention and inhibition [19]. Taken together, these measures suggest that some ADHD traits may intersect with curiosity traits such as novelty seeking and exploration, even if the underlying mechanisms differ.

Clarifying whether and how curiosity relates to ADHD traits has both theoretical and practical implications. Theoretically, it may help refine our understanding of the broader ADHD phenotype beyond known functional difficulties. Practically, identifying whether ADHD traits are associated with curiosity could inform more targeted approaches to supporting curiosity-driven engagement in educational and occupational settings. However, empirical research on the relationship between ADHD and curiosity remains limited. The present study addresses this gap by examining whether ADHD trait scores are associated with overall curiosity levels. Due to limited prior quantitative research examining the relationship between ADHD and curiosity, no directional hypothesis was specified. Instead, the present study aimed to examine whether ADHD traits are associated with individual differences in curiosity in adults.

## Methods

This study employed a cross-sectional survey design to examine the relationship between ADHD traits and curiosity in adults.

### Participants

Adults aged 18 years and older were eligible to participate if they were fluent in English, resided in the United Kingdom, and were able to provide informed consent. Including participants across diagnostic statuses allowed us to examine whether associations between ADHD traits and curiosity reflect diagnosis-specific or broader dimensional patterns. Participants were recruited through convenience sampling using multiple channels: the university research circular, social media platforms, and academic newsletters. Because the study was openly described as investigating ADHD traits and curiosity, recruitment materials were likely to attract individuals with a personal connection to ADHD. Recruitment included posts shared via the laboratory’s social media channels and newsletter, which is likely to have contributed to the high proportion of participants reporting an ADHD diagnosis.

Recruitment materials outlined the study purpose, eligibility requirements, estimated completion time, and the voluntary and anonymous nature of participation. Participants were offered the option to enter a prize draw to win one of three shopping vouchers (£100, £50, or £25). Entry into the draw was voluntary and completed via a separate form that is not linked to survey responses, in order to maintain anonymity.

Given previous work showing small associations between hyperactivity–impulsivity and curiosity [9], an a priori power analysis was conducted using G*Power 3.1 [20] based on a small incremental effect (*f*² = 0.05). For a multiple regression including five predictors, this indicated that a minimum sample of 262 participants was required to achieve 95% power at *α* = 0.05. Data collection remained open for one month during which a total of 521 participants completed the survey.

### Measures

Participants provided background information including age, gender, ethnicity, education level, employment status, ADHD diagnosis status (formal clinical diagnosis, self-diagnosis, or no diagnosis), current medication use for ADHD, and any additional neurodevelopmental conditions such as autism, dyslexia, dyscalculia, or other. The survey questions were reviewed by members of the project’s research advisory board, which includes individuals with lived experience of ADHD to ensure appropriate language and clarity.

ADHD traits were assessed using the Adult ADHD Self-Report Scale version 1.1 (ASRS-v1.1), an 18-item self-report measure developed by the World Health Organization [21]. The scale evaluates the frequency of ADHD traits across two domains: inattention (9 items) and hyperactivity–impulsivity (9 items). Participants rated each item on a 5-point Likert scale ranging from 0 (Never) to 4 (Very Often). Total and subscale scores were obtained by summing the relevant items, with higher scores indicating more frequent ADHD-related behaviors.

Curiosity was measured using the Curiosity and Exploration Inventory-II (CEI-II), a 10-item self-report instrument comprising two 5-item subscales [17]. The CEI-II was selected because it provides a parsimonious, well-validated measure of general trait curiosity that directly captures exploratory motivation and engagement with novelty while avoiding the added complexity of multidimensional curiosity scales. Items are rated on a 5-point scale from 1 (Very slightly or not at all) to 5 (Extremely). The ‘stretching’ subscale (5 items) assesses motivation to seek out new knowledge and experiences, while the ‘embracing’ subscale (5 items) measures willingness to engage with novelty and uncertainty. Total and subscale scores were calculated as the sum of their constituent items, with higher scores reflecting greater curiosity.

### Procedure

An anonymous online questionnaire was administered via Qualtrics (Provo, UT). After accessing the survey link, participants viewed an information sheet and provided electronic informed consent via a series of checkbox statements before proceeding to the main questionnaires. No personal identifiers were collected, and participants could withdraw at any point before submission by closing their browser window. However, withdrawal after submission was not possible due to the anonymous nature of the data. Upon completion, participants could optionally enter a prize draw for one of three shopping vouchers (£100, £50, or £25) by accessing a separate, unlinked form to maintain anonymity.

### Data analysis

All analyses were conducted using Jamovi (version 2.6). Descriptive statistics were computed for all variables, and internal consistency was evaluated using Cronbach’s alpha [22] for each scale composite. Survey completion required responses to all ASRS-v1.1 and CEI-II items and were enforced through the survey platform, so no missing data were present on these measures in the final sample. Data distributions were examined using skewness and kurtosis statistics along with Q-Q plots to assess normality assumptions.

All statistical tests were two-tailed with significance set at *α* = 0.05. Pearson’s correlations were calculated to examine associations between ADHD traits (total symptoms, inattention, hyperactivity–impulsivity) and curiosity dimensions (total curiosity, stretching, embracing). A Bonferroni correction [23] was applied to adjust for multiple comparisons, with 95% confidence intervals reported for correlations and key group comparisons.

To determine whether ADHD traits uniquely predicted curiosity, a multiple linear regression was conducted with total curiosity (CEI-II Total) as the outcome variable and inattention and hyperactivity–impulsivity as predictors, controlling for age, gender, and education level. Age, gender, and education level were included as covariates based on their established or theoretically plausible associations with curiosity [17]. Because the number of participants identifying as non-binary or another gender was too small for reliable multi-category estimation, gender was entered as a binary covariate (woman/man) in the regression. Accordingly, participants identifying as non-binary or another gender were excluded from this model. Ethnicity and employment status were not included because there is no established theoretical or empirical basis linking these variables to trait curiosity as measured by the CEI-II, and the uneven distribution of categories for both variables would have limited the reliability of their estimation. Co-occurring neurodivergent conditions and medication status were also not included in the primary model. Neurodivergent conditions such as autism were recorded as binary self-report variables rather than validated dimensional measures, limiting their interpretive value as covariates. Medication status was captured as a single binary variable that does not account for variability in medication type, dosage, adherence, or timing relative to survey completion, and is further confounded with ADHD severity and diagnostic status. However, supplementary analyses were conducted to verify that the primary pattern of results was robust to the inclusion of autism status and medication use as additional covariates.

Lastly, a contingency analysis examined whether elevated hyperactivity–impulsivity and elevated curiosity co-occurred more often than expected. Participants scoring at least one standard deviation above the sample mean on hyperactive–impulsive symptoms and on total curiosity were classified as high on each dimension. This analysis complements the regression by examining whether high hyperactivity–impulsivity and high curiosity cluster together at the distributional tails, a pattern that cannot be inferred from linear associations alone.

## Results

### Sample characteristics

A total of 521 participants completed the survey (Table 1). Participants ranged in age from 18 to 86 years (*M* = 35.3, *SD* = 12.2), with a median age of 33 years. The gender distribution was predominantly women (72.9%), followed by men (22.3%), with a smaller proportion identifying as non-binary (4.4%) or another gender (0.4%). Ethnic composition was majority White (73.3%), with additional representation from Asian or Asian British (16.3%), Black, Black British, Caribbean, or African participants (1.2%), mixed or multiple ethnic groups (5.0%), and other ethnic backgrounds (4.2%).

**Table 1:** Participant characteristics

Variable	Category	*n* (%)
Gender	Man	116 (22.3%)
	Woman	380 (72.9%)
	Non-binary	23 (4.4%)
	Other	2 (0.4%)
Ethnicity	Asian or Asian British	85 (16.3%)
	Black, Black British, Caribbean or African	6 (1.2%)
	White	382 (73.3%)
	Mixed or Multiple ethnic groups	26 (5.0%)
	Other ethnic background	22 (4.2%)
Diagnosis	Diagnosed in a healthcare setting	264 (50.7%)
	Diagnosed in an educational setting	13 (2.5%)
	Self-diagnosed	109 (20.9%)
	No diagnosis	135 (25.9%)
Medication	Yes, currently taking medication for ADHD	151 (29.0%)
	No, not currently taking medication for ADHD	370 (71.0%)
Education	Postgraduate degree (e.g., MA, MSc, PhD)	256 (49.1%)
	Undergraduate degree (e.g., BA, BSc)	186 (35.7%)
	Further education (e.g., A-levels, BTEC, NVQ)	53 (10.2%)
	Secondary education (e.g., GCSEs, O-levels)	9 (1.7%)
	Other professional or vocational qualification	15 (2.9%)
	No formal qualifications	2 (0.4%)
Employment	Student	128 (24.6%)
	Employed full-time	198 (38.0%)
	Employed part-time	67 (12.9%)
	Self-employed (e.g., entrepreneur, freelancer)	78 (15.0%)
	Unemployed and seeking work	39 (7.5%)
	Retired	11 (2.1%)

Just over half of the sample (50.7%) reported a formal ADHD diagnosis obtained through a healthcare provider (e.g., NHS, private psychiatrist), 2.5% reported receiving a diagnosis in an educational setting (e.g., school, university disability services, educational psychologist), 20.9% identified as self-diagnosed while awaiting or pursuing formal assessment, and 25.9% reported no diagnosis. In addition, 151 participants (28.1%) reported at least one other neurodivergent condition. The most frequently reported condition was autism (*n* = 76), followed by dyslexia (*n* = 36) and dyscalculia (*n* = 15). Because participants could report multiple conditions, these categories are not mutually exclusive. The remaining participants did not report any known other neurodivergent conditions. Regarding medication status, 29.0% of the total sample reported taking ADHD-related medication, while 71.0% were not medicated at the time of the survey.

The sample was highly educated. Nearly half (49.1%) reported holding a postgraduate degree, and a further 35.7% held an undergraduate degree. Smaller proportions reported further education qualifications (10.2%), vocational or professional qualifications (2.9%), secondary education (1.7%), or no formal qualifications (0.4%). Employment status was varied: 38.0% were employed full-time, 12.9% part-time, and 15.0% self-employed. Students comprised 24.6% of the sample, while 7.5% were unemployed and seeking work, and 2.1% were retired.

### Descriptive statistics

Descriptive statistics were calculated for all ASRS and CEI-II scale scores. ADHD trait scores had a mean of 47.7 (*SD* = 11.8) for the total scale, with means of 25.2 (*SD* = 5.99) for inattention and 22.4 (*SD* = 6.61) for hyperactivity–impulsivity. Curiosity scores had a mean of 34.5 (*SD* = 7.83) for the total scale, with means of 19.0 (*SD* = 3.94) for Stretching and 15.5 (*SD* = 4.68) for Embracing. A total curiosity score of 40.0 corresponded to the 75th percentile of the distribution.

Internal consistency was examined for all ADHD and curiosity measures. The ASRS-18 total score demonstrated excellent reliability (*α* = 0.90). The inattention and hyperactivity–impulsivity subscales also showed good reliability, with *α* = 0.82 and *α* = 0.84, respectively. The CEI-II total score showed strong internal consistency (*α* = 0.87). Reliability for the two CEI-II subscales was also acceptable, with *α* = 0.80 for the Stretching subscale and *α* = 0.82 for the Embracing subscale. Overall, these coefficients indicate robust internal consistency across all scales.

### Correlations between ADHD traits and curiosity

Pearson’s correlations were computed to explore potential associations between ADHD (ASRS-18) and curiosity (CEI-II) traits (Table 2). ADHD trait scores were positively correlated with all curiosity measures. Correlations were small-to-moderate in magnitude, ranging from *r* = .22 to *r* = .30, and all remained statistically significant after Bonferroni correction. The strongest associations were observed between the hyperactivity–impulsivity subscale and curiosity measures, while inattention showed slightly weaker correlations across all curiosity dimensions. Although the inattention and hyperactivity–impulsivity subscales were strongly correlated (*r* = .76), variance inflation factors (VIFs = 2.42 and 2.48) indicated acceptable levels of multicollinearity.

**Table 2:** Pearson’s correlation matrix

	Total ADHD	Inattentive	Hyperactive –Impulsive
Total Curiosity	0.29*** [0.21, 0.36]	0.24*** [0.15, 0.32]	0.30*** [0.22, 0.38]
Stretching	0.27*** [0.18, 0.34]	0.22*** [0.13, 0.30]	0.28*** [0.20, 0.36]
Embracing	0.26*** [0.18, 0.34]	0.21*** [0.13, 0.29]	0.27*** [0.19, 0.35]

* *p* < .05, ** *p* < .01, *** *p* < .001. *p* values are Bonferroni-adjusted for multiple comparisons

### Group differences by diagnostic status

Welch’s one-way ANOVAs were conducted to examine differences in ADHD trait scores across diagnostic status (formally diagnosed ADHD in healthcare or educational settings, self-identified ADHD, and no ADHD diagnosis). Significant effects of diagnostic status were observed for total ADHD trait scores, *F*(2, 233) = 88.30, *p* < .001, inattention, *F*(2, 230) = 90.60, *p* < .001, and hyperactivity–impulsivity, *F*(2, 240) = 67.10, *p* < .001. Games–Howell post-hoc tests indicated that both formally diagnosed and self-identified participants reported substantially higher ADHD trait scores across all domains compared with those reporting no diagnosis (all *p* < .001), with large effect sizes. For total ADHD traits, formally diagnosed (*M* = 52.10, *SD* = 8.33, *d* = 1.60) and self-identified participants (*M* = 50.70, *SD* = 8.31, *d* = 1.33) scored significantly higher than those with no diagnosis (*M* = 36.10, *SD* = 12.75). The same pattern was observed for inattention (formally diagnosed: *M* = 27.60, *SD* = 4.06, *d* = 1.65; self-identified: *M* = 26.60, *SD* = 4.14, *d* = 1.30; no diagnosis: *M* = 19.30, *SD* = 6.57) and hyperactivity–impulsivity (formally diagnosed: *M* = 24.50, *SD* = 5.29, *d* = 1.31; self-identified: *M* = 24.00, SD = 5.20, *d* = 1.16; no diagnosis: *M* = 16.80, *SD* = 6.90). No significant differences were observed between formally diagnosed and self-identified participants across any domain. On this basis, these groups were combined into a single ADHD group for subsequent analyses.

To examine whether curiosity differed at a broader diagnostic level, curiosity scores were compared between participants in the combined ADHD group and those reporting no ADHD diagnosis. Welch’s independent-samples *t*-tests indicated that participants with ADHD reported moderately higher total curiosity than those without an ADHD diagnosis, *t*(209) = 3.93, *p* < .001, *d* = 0.41. Similar differences were observed for the Stretching, *t*(208) = 3.91, *p* < .001, *d* = 0.41, and Embracing subscales, *t*(214) = 3.30, *p* = .001, *d* = 0.34.

### Regression analyses

A multiple regression examined whether inattention and hyperactivity–impulsivity predicted curiosity after demographic variables were taken into account. The overall model was significant, *F*(5, 490) = 17.20, *p* < .001, and explained 14.9% of the variance in curiosity. Of the two ADHD symptom domains, only hyperactivity–impulsivity significantly predicted curiosity (*β* = 0.26, *p* < .001); inattention was not a significant predictor (*p* = .459). Age (*β* = 0.15, *p* < .001) and education level (*β* = 0.18, *p* < .001) were also positive predictors, while gender was not significant (*p* = .116). Although variance inflation factors indicated acceptable multicollinearity, supplementary regressions were conducted to model each ADHD symptom domain separately. Inattention (*β* = 0.31, *p* < .001) and hyperactivity–impulsivity (*β* = 0.34, *p* < .001) each predicted curiosity when entered alone, but only hyperactivity–impulsivity remained significant in the combined model, indicating that it was the only symptom domain to uniquely explain variance in curiosity. Running the regression separately within ADHD and non-ADHD groups produced the same pattern of results: hyperactivity–impulsivity significantly predicted curiosity in both groups (non-ADHD: *β* = 0.27, *p* = .047; ADHD: *β* = 0.20, *p* = .001), whereas inattention did not (non-ADHD: *β* = 0.04, *p* = .783; ADHD: *β* = 0.03, *p* = .628). The coefficient in the non-ADHD group did not significantly differ from the ADHD group coefficient (*z* = 0.24, *p* = .809), indicating that the association between hyperactivity–impulsivity and curiosity was stable across diagnostic status. Taken together, these results show that hyperactive–impulsive symptoms, but not inattentive symptoms, uniquely predicted curiosity after demographic factors were controlled.

Autism status and medication use were not included in the primary model for several reasons. Autism status was based on self-report of a co-occurring condition rather than a validated measure of autistic traits, limiting its interpretive value as a covariate. Similarly, medication status was captured as a binary variable that does not account for variability in medication type, dosage, adherence, or timing relative to survey completion, and is confounded with ADHD severity and diagnostic status, making its effects difficult to interpret independently. However, given that both variables represent non-negligible subgroups within the sample, a supplementary regression was conducted to assess whether the primary pattern of results was robust to their inclusion. The pattern of results was unchanged: hyperactivity–impulsivity remained a significant predictor of curiosity (*β* = 0.23, *p* < .001), while inattention (*β* = 0.07, *p* = .278) and medication status (*β* = 0.07, *p* = .130) were not significant. Autism status was associated with lower curiosity (*β* = −0.13, *p* = .002), indicating that co-occurring autism did not inflate the observed association between hyperactivity–impulsivity and curiosity.

Lastly, a robustness analysis in which conceptually overlapping items were removed from both the ASRS and CEI-II produced the same pattern: hyperactivity–impulsivity remained a significant predictor of pruned curiosity scores (*β* = 0.22, *p* < .001), whereas inattention did not (*p* = .478). Full details of both those robustness checks are reported in the Supplementary Materials.

### Contingency analysis

To further explore whether elevated hyperactive–impulsive symptoms co-occur with particularly high levels of curiosity, a contingency analysis was conducted. Participants scoring at least one standard deviation above the sample mean on hyperactivity–impulsivity (ASRS-HI ≥ 29) and total curiosity (CEI-II ≥ 42) were classified as high on each dimension. Overall, 8.1% of the sample met criteria for both high hyperactivity–impulsivity and high curiosity. High curiosity was more prevalent among participants with elevated hyperactive–impulsive symptoms (41.6%) than among those below this threshold (16.2%), *χ*²(1, *N* = 521) = 31.5, *p* < .001. It should be noted that this subgroup was defined using distribution-based thresholds rather than clinical cut-offs, and the analysis is intended to illustrate distributional patterns rather than to identify a discrete category.

## Discussion

Existing work has linked the broader ADHD phenotype to traits such as reward sensitivity, novelty seeking, and heightened approach behavior [24–26], and qualitative studies have highlighted curiosity as a recurrent theme in how adults with ADHD describe their everyday experiences, often framing it as both a strength and a challenge [6, 7, 10, 12]. However, direct examinations of the relationship between curiosity and ADHD traits remain limited. This cross-sectional study examined associations between ADHD traits and curiosity in an adult sample, showing small-to-moderate positive correlations between self-reported ADHD traits and curiosity. Curiosity was moderately higher among participants with ADHD than those without an ADHD diagnosis, although diagnostic status alone did not account for individual differences in curiosity once ADHD symptom dimensions were considered. Of the two ADHD symptom dimensions included in the regression model, only hyperactive–impulsive symptoms uniquely predicted curiosity once demographic factors were controlled, whereas inattentive symptoms were not a significant predictor. Age and education were also significant positive predictors of curiosity, while gender was not. The association between hyperactivity–impulsivity and curiosity was robust to the inclusion of autism status and medication use as covariates in a supplementary analysis. Autism status was associated with lower curiosity, suggesting that co-occurring autism did not inflate the observed associations.

Several explanations may account for the positive associations between ADHD traits and curiosity. One possibility is construct overlap, where items in the ASRS and CEI-II capture partly similar tendencies – for example, novelty seeking or openness to change. If this overlap were substantial, we would expect similar correlation magnitudes across ADHD dimensions, which is broadly the case, though hyperactivity–impulsivity shows a slightly stronger association. Prior work has shown that curiosity is moderately correlated with broader personality traits linked to exploration, including openness to experience and novelty seeking [16–18], which themselves show small-to-moderate associations with ADHD traits, particularly hyperactivity–impulsivity [19, 27]. However, the constructs are conceptually distinct: ADHD symptom measures relate to behavioral regulation and attentional control, whereas curiosity as measured by the CEI-II is understood as a motivational orientation toward exploration and engagement with novelty and uncertainty. If construct overlap were the primary driver of the observed associations, similar effect sizes would be expected across inattentive and hyperactive–impulsive dimensions. Instead, hyperactivity–impulsivity showed a consistently stronger relationship with curiosity, suggesting that shared variance alone is unlikely to fully explain the results. This interpretation is further supported by a robustness analysis in which conceptually overlapping items were removed from both scales, which produced the same pattern of results.

A second explanation is that ADHD-related impulsivity and curiosity may draw on overlapping motivational and reward processes. Hyperactive–impulsive symptoms are associated with heightened responsiveness to immediate rewards and a greater tendency to initiate action quickly [28, 29]. Curiosity, as measured by the CEI-II scale, similarly reflects a motivation to engage with novel or uncertain situations [17]. Neurocognitive models describe substantial overlap between curiosity and impulsivity, with both involving frontostriatal networks and dopaminergic midbrain pathways [14], networks that are also central to theories of reward-driven behavior in ADHD [30–33]. In line with those models, hypercuriosity has been proposed as an intensified and difficult-to-inhibit desire to explore and seek information, characterised by both frequent attentional shifts toward novel stimuli and, in some contexts, deep absorption when curiosity is strongly engaged [15]. Our finding that hyperactive–impulsive symptoms uniquely predicted curiosity is consistent with this theoretical account, as is the contingency analysis showing that high hyperactivity–impulsivity and high curiosity co-occurred more often than expected. Although the present study does not examine mechanisms directly, this pattern is compatible with the hypothesis that stronger hyperactive–impulsive tendencies may amplify responsiveness to information-rich stimuli, contributing to increased exploratory behavior.

A third explanation concerns self-interpretation of behavior. Individuals with higher ADHD traits may interpret tendencies such as shifting focus or acting quickly as expressions of curiosity. This subjective framing could contribute to modest correlations between self-reported traits and curiosity and aligns with a growing body of research highlighting potential strengths associated with neurodivergent profiles, in particular qualitative research showing that individuals with ADHD often describe curiosity as both a challenge and an asset, driving deep engagement in areas of interest while sometimes complicating task prioritization [6, 7, 10]. The regression finding that hyperactivity–impulsivity uniquely predicted curiosity after controlling for demographic factors is consistent with a substantive association, but because both constructs were measured via self-report, the possibility that shared self-perceptual framing contributed to this pattern cannot be ruled out.

A final explanation is that ADHD traits and curiosity may simply co-vary as distinct but related dispositions. The small-to-moderate correlations across ADHD dimensions and curiosity suggest that the constructs are connected without being redundant. At the same time, the regression results indicate that hyperactivity–impulsivity carries some unique variance in curiosity that is not shared with inattentive symptoms or demographic factors. As noted above, the robustness analysis further supports this interpretation. Taken together, this pattern is consistent with the view that ADHD traits and curiosity overlap to some extent – particularly through motivational tendencies reflected in hyperactivity–impulsivity – while remaining conceptually and empirically distinct [15].

The present finding that hyperactivity–impulsivity, but not inattention, uniquely predicted curiosity aligns with emerging quantitative evidence suggesting that curiosity relates more strongly to the hyperactive–impulsive dimension of the ADHD phenotype [9]. The association between hyperactivity–impulsivity and curiosity was not significantly different between diagnostic groups, further supporting the dimensional nature of this relationship. Consistent with the motivational account outlined above, hyperactive–impulsive symptoms reflect heightened behavioral activation and approach-oriented responses that overlap with the exploratory drive captured by curiosity measures [16]. In contrast, inattentive symptoms primarily reflect difficulties with sustaining focus and regulating attention – challenges that are distinct from the motivational pull toward novelty and uncertainty that characterises curiosity. As such, hyperactivity–impulsivity may be more closely tied to the cognitive and behavioral processes that support curiosity, whereas inattention does not uniquely account for curiosity once other factors are considered.

These findings also connect to broader accounts of how impulsive tendencies may be adaptive in certain contexts. The tendency to act quickly in response to salient cues may make exploratory behavior more likely [34], and models suggest that such impulsive behavior might be functional in unpredictable environments where access to resources is likely to be interrupted [35]. From this perspective, resolving uncertainty or gaining new information may be especially rewarding for individuals who are more driven by immediate, salient outcomes [36, 37].

The findings contribute to a growing body of work that considers potential strengths alongside difficulties associated with ADHD. Qualitative and quantitative research highlights themes such as curiosity, energy, creativity, and willingness to take risks, which some adults and young people with ADHD experience as resources in supportive environments [8, 10, 38–41]. The present results are consistent with this literature: the observation that hyperactive–impulsive traits – typically associated with regulatory difficulties – were positively associated with self-reported curiosity suggests that some traits commonly framed as challenges may also relate to tendencies that individuals experience as strengths. This association opens the door to further investigation of how traits within the ADHD phenotype may relate to broader personality and motivational dimensions [9, 27, 42–48]. For instance, in some individuals with elevated hyperactive–impulsive symptoms, the same tendencies that produce restlessness in constrained environments might, under conditions that allow exploration, translate into deeper intellectual engagement. However, the present study did not examine functional outcomes, and further research is needed to determine whether and when curiosity associated with hyperactive–impulsive traits translates to advantages in educational or occupational settings.

A further implication concerns self-perceptions and identity. People with ADHD are frequently exposed to public stigma and negative stereotypes, and internalised stigma is common and associated with lower self-esteem, greater functional impairment, and poorer quality of life [49–51]. Behaviors such as rapid task-shifting, frequent questioning, or seeking stimulation may clash with classroom expectations for sustained focus on routine material, potentially reinforcing negative feedback cycles and undermining academic self-concept [52–54]. At the same time, research on self-perceptions suggests that strength-based perspectives can buffer against some adverse outcomes through self-understanding, identity-affirmation, and positive reappraisal of one’s differences, which are associated with greater resilience and improved mental health [6, 55, 56]. Recognising that hyperactive–impulsive traits can relate to exploratory drive and curiosity – not only to challenges – may help support a more positive sense of identity, and viewing curiosity as a motivating force rather than solely a source of distraction could also inform teaching, coaching, career guidance, and workplace accommodations that support curiosity.

However, several limitations should be considered when interpreting these findings. First, the study relied on a cross-sectional design, which prevents causal inferences. The observed associations could reflect influences of ADHD traits on curiosity, effects of curiosity on ADHD-related behaviors, or shared underlying factors. Longitudinal and experimental designs would allow stronger tests of directionality. In addition, all measures were self-reported. Although the instruments used have strong psychometric support, self-report introduces the possibility of shared method variance, recall biases, and influences of participants’ interpretations of their own behavior [57, 58]. Future research would benefit from combining self-report with behavioral indices of curiosity, along with clinician-rated or multi-informant assessments of ADHD traits. Online recruitment may also have attracted individuals with particular interest in or awareness of ADHD, and the transparency of the study aims may have shaped how participants interpreted and reported their experiences [59–61]. Additionally, voluntary participation in an online survey may itself select for higher curiosity, as individuals who choose to engage with research opportunities may be more curious than those who do not. This self-selection could have inflated curiosity scores across the sample and may limit the generalisability of the reported mean levels of curiosity, though it is less likely to account for the observed within-sample associations between ADHD trait dimensions and curiosity. More broadly, there are likely to be other factors relevant to the relationship between ADHD traits and curiosity such as personality traits beyond those captured here that were either not measured or could not be meaningfully included given the constraints of the available data. Future research would benefit from incorporating a wider range of potential confounders and moderators to refine our understanding of this association.

Finally, features of the sample limit generalisability. Participants were predominantly female, White, and highly educated, which may limit the extent to which the findings apply to groups that differ in gender distribution, socioeconomic background, ethnicity, or cultural context. In particular, the high proportion of participants with an ADHD diagnosis likely reflects targeted dissemination through ADHD-related online communities, and the sample should not be considered representative of the general population. The regression analysis also excluded participants identifying as non-binary or another gender because these groups were too small for stable multi-category modelling. As a result, the study cannot evaluate whether associations between ADHD traits and curiosity vary across gender identities beyond the binary woman/man categories. This is noteworthy given emerging research suggesting that gender-diverse individuals may experience neurodivergence and related traits in distinct ways [62–64]. Despite these limitations, the study’s large sample, use of well-validated dimensional measures, and convergence across analytic approaches support the reliability of the associations observed in this study and provide a foundation for future research.

Future research should examine whether the observed patterns replicate in more diverse samples, including those recruited through clinical services and in cultural contexts where curiosity may be expressed or valued differently. Longitudinal studies are also needed to track how curiosity and ADHD traits change over time and to assess whether hypercuriosity is linked to functional outcomes in some environments, including whether it is associated with differences in learning, work, or wellbeing. Neurocognitive research could further clarify the mechanisms of hypercuriosity in ADHD, particularly work examining reward sensitivity and dopaminergic function, given their relevance to both curiosity and impulsivity [14, 15, 65, 66]. Combining neural, behavioral, and self-report measures would provide a more comprehensive understanding of how these systems interact. Intervention studies could test whether approaches that deliberately leverage curiosity – such as learning environments that offer autonomy, novelty, or opportunities for exploration – improve engagement or performance for individuals with ADHD and help inform the design of more supportive and inclusive educational and occupational settings. Finally, cultural norms shape how curiosity is expressed, encouraged, or constrained [67, 68]. Research with more diverse samples and cross-cultural studies is needed to determine whether associations between ADHD traits and curiosity hold across demographic groups and cultural contexts.

## Conclusion

This study provides initial evidence that ADHD traits, and hyperactive–impulsive symptoms in particular, may be associated with higher self-reported trait curiosity in adults. This pattern is consistent with theoretical accounts of hypercuriosity and suggests that this construct may be relevant for understanding how some individuals with ADHD interact with their environments. These findings contribute to a broader understanding of ADHD that considers not only difficulties with regulation but also tendencies related to engagement with novelty and uncertainty. Although the study does not address functional outcomes, it provides a foundation for examining when and under what conditions curiosity associated with hyperactive–impulsive traits relates to learning, work, or wellbeing. Future research should clarify the mechanisms underlying this association and investigate whether these findings have practical implications for educational and occupational settings.

## Supplementary Information

Supplementary Material 1.

## Data Availability

The datasets generated and analysed during the current study are available in the King’s Open Research Data System (KORDS) repository: 10.18742/31070851.
