# Supplementary material for: Hyperactive–impulsive ADHD traits predict higher curiosity in adults: evidence from a cross-sectional study
Source: BMC Psychol. 2026 Apr 10;14:723. doi: 10.1186/s40359-026-04504-7 (PMC13181895; doi:10.1186/s40359-026-04504-7)
Supplement: Supplementary file 1 — Supplementary Material 1. [file 40359_2026_4504_MOESM1_ESM.pdf]

Supplementary Materials

Table S1. Descriptive statistics by diagnostic group.

	Group	Age	Education	ASRS Total	ASRS_I	ASRS_HI	CEI Total	CEI Stretch	CEI Embrace
N	**ADHD**	386	386	386	386	386	386	386	386
	**non-ADHD**	135	135	135	135	135	135	135	135
**Mean**	**ADHD**	36.8	4.23	51.7	27.3	24.4	35.4	19.4	16.0
	**non-ADHD**	31.0	4.32	36.1	19.3	16.8	32.1	17.8	14.4
**Median**	**ADHD**	35.0	4.0	51.0	27.5	24.0	35.0	20.0	16.0
	**non-ADHD**	27	5.0	38.0	19.0	17.0	33.0	19.0	15.0
SD	**ADHD**	12.0	0.99	8.34	4.10	5.26	7.41	3.72	4.49
	**non-ADHD**	11.8	0.84	12.7	6.57	6.90	8.51	4.31	5.01
**Min.**	**ADHD**	18	0	28.0	14.0	11.0	11.0	6.00	5.00
	**non-ADHD**	18	1	0.00	0.00	0.00	13.0	7.00	5.00
**Max.**	**ADHD**	86	5	72.0	36.0	36.0	50.0	25.0	25.0
	**non-ADHD**	68	5	68.0	33.0	36.0	48.0	25.0	24.0

Figure S1. Distribution of total ADHD trait scores (ASRS-18) and total curiosity scores (CEI-II) by diagnostic group.

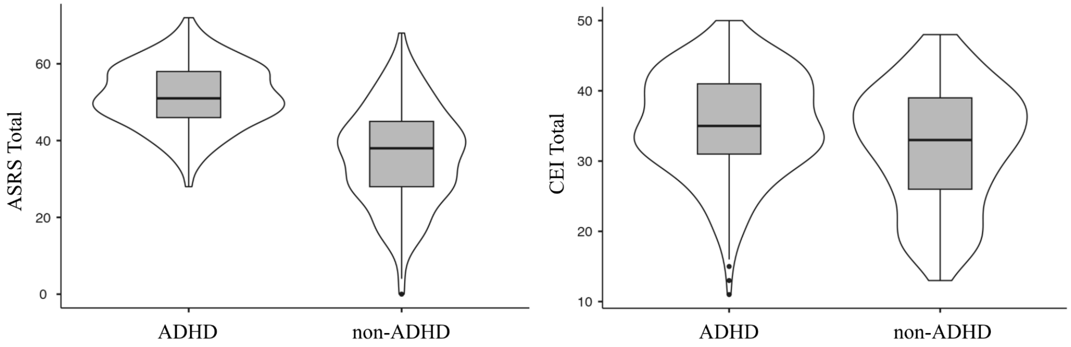

Table S2. Pearson's correlation matrix for all continuous study variables.

	1	2	3	4	5	6	7	8
**1. Age**	—							
**2. Education**	-0.033	—						
**3. ASRS Total**	0.190***	-0.087*	—					
**4. ASRS_I**	0.150***	-0.099*	0.932***	—				
**5. ASRS_HI**	0.205***	-0.066	0.944***	0.761***	—			
**6. CEI Total**	0.206***	0.139**	0.287***	0.236***	0.300***	—		
**7. CEI Stretch**	0.213***	0.151***	0.265***	0.217***	0.278***	0.891***	—	
**8. CEI Embrace**	0.165***	0.105*	0.257***	0.212***	0.268***	0.924***	0.649***	—

Note. * $p < .05$, ** $p < .01$, *** $p < .001$

Figure S2. Scatterplot of hyperactivity–impulsivity (ASRS-HI) and total curiosity (CEI-II) by diagnostic group.

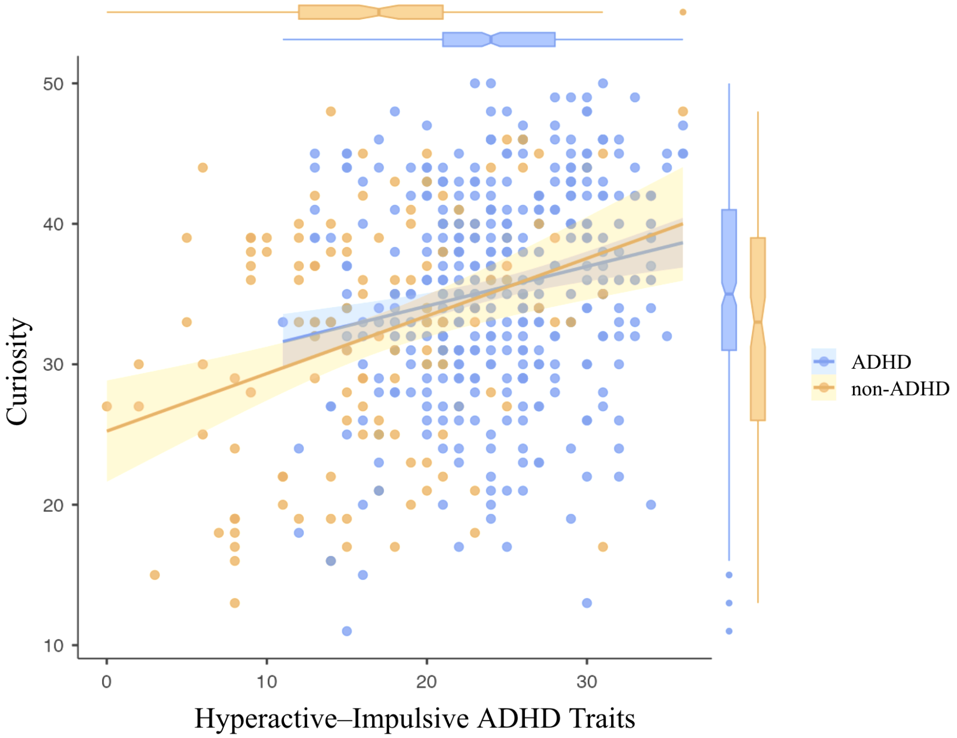

Note. Bivariate association between hyperactivity–impulsivity scores and total curiosity, with separate regression lines and 95% confidence bands by diagnostic group. The positive association was observed in both groups, consistent with the non-significant difference between group regression coefficients reported in the main text ($z = 0.24$, $p = .809$).

Robustness Analysis: Construct Overlap

To assess whether the observed association between ADHD traits and curiosity was driven by overlapping item content, a robustness analysis was conducted using pruned versions of both scales. Four ASRS items reflecting high motor activity and restlessness were removed (items 5, 6, 13, and 14: fidgeting/squirming, feeling driven by a motor, feeling restless or fidgety, and difficulty unwinding or relaxing), as these most closely overlap with the novelty-seeking and stimulation-oriented content of the CEI-II. The remaining 14 ASRS items were retained. On the CEI-II side, five items from the Embracing subscale were removed (items 2, 4, 6, 8, and 10: enjoying uncertainty, seeking new experiences, liking things that are frightening, preferring unpredictable jobs, and embracing unfamiliar situations), as these most directly capture novelty seeking and tolerance for unpredictability — content that overlaps conceptually with hyperactive–impulsive behaviours. The remaining five Stretching items, which capture more cognitive and growth-oriented aspects of curiosity, were retained.

Both pruned scales showed good internal consistency (ASRS pruned: $\alpha = .885$; CEI-II pruned: $\alpha = .803$). The correlation between pruned ADHD traits and pruned curiosity remained positive and significant, though slightly attenuated ($r = .228$, $p < .001$) compared to the original ($r = .287$). A multiple regression using the pruned curiosity score as the outcome variable, with inattention and hyperactivity–impulsivity as predictors alongside age, gender, and education, produced the same pattern as the primary analysis: the overall model was significant ($R^2 = .135$, $F(5, 490) = 15.3$, $p < .001$), hyperactivity–impulsivity remained a significant predictor ($\beta = .22$, $p < .001$), and inattention did not ($\beta = .05$, $p = .478$).

Table S3. Pruned ASRS reliability analysis.

Scale Reliability Statistics
(14 items)

	Cronbach's α
**Scale**	0.885

Table S4. Pruned CEI reliability analysis.

Scale Reliability Statistics
(5 items)

	Cronbach's α
**Scale**	0.803

Table S5. Correlation between pruned ASRS and pruned CEI-II scores.

		ASRS_pruned
**CEI_pruned**	Pearson's *r*	0.228
	p-value	<.001

Table S6. Model fit for multiple regression predicting pruned curiosity scores.

Model	*R*	R^2
1	0.367	0.135

Note. Models estimated using sample size of *N*=496.

Table S7. Regression coefficients predicting pruned curiosity scores (CEI-II pruned).

			95% Confidence Interval					95% Confidence Interval	
**Predictor**	**Estimate**	**SE**	**Lower**	**Upper**	***t***	***p***	**Stand. Estimate**	**Lower**	**Upper**
Intercept	10.6408	1.3139	8.0593	13.2222	8.099	<.001			
ASRS_I	0.0304	0.0428	-0.0538	0.1146	0.710	0.478	0.0464	-0.0821	0.1749
ASRS_HI	0.1327	0.0393	0.0554	0.2100	3.372	<.001	0.2233	0.0932	0.3534
Gender	-0.2434	0.4003	-1.0299	0.5430	-0.608	0.543	-0.0259	-0.1097	0.0578
Education	0.7440	0.1750	0.4001	1.0878	4.251	<.001	0.1810	0.0974	0.2647
Age	0.0530	0.0141	0.0254	0.0807	3.766	<.001	0.1629	0.0779	0.2478

Autism Status and Medication Use as Additional Covariates

To assess whether the primary pattern of results was robust to the inclusion of autism status and medication use, a supplementary multiple regression was conducted adding these two variables as covariates alongside the predictors and covariates from the primary model. Autism status was coded as a binary variable based on self-reported co-occurring autism. Medication status was coded as a binary variable indicating whether the participant was currently taking medication for ADHD. Results are summarised in the main text; full model output is reported in the following table.

Table S8. Model fit and regression coefficients for multiple regression predicting curiosity with autism status and medication use as additional covariates.

Model Fit Measures

				Overall Model Test			
**Model**	***R***	***R²***	**Adjusted *R²***	**F**	***df*1**	***df*2**	***p***
1	0.412	0.170	0.157	13.7	7	470	<.001

Note. Models estimated using sample size of *N*=478.

			95% Confidence Interval					95% Confidence Interval	
**Predictor**	**Estimate**	**SE**	**Lower**	**Upper**	***t***	***p***	**Stand. Estimate**	**Lower**	**Upper**
Intercept	18.9144	2.6053	13.7950	24.034	7.26	<.001			
ASRS I	0.0943	0.0869	-0.0765	0.265	1.08	0.278	0.0731	-0.0593	0.2055
ASRS HI	0.2723	0.0792	0.1166	0.428	3.44	<.001	0.2332	0.0999	0.3666
Gender	-1.0625	0.8058	-2.6458	0.521	-1.32	0.188	-0.0568	-0.1415	0.0279
Education	1.4009	0.3485	0.7160	2.086	4.02	<.001	0.1719	0.0879	0.2559
Age	0.0908	0.0282	0.0353	0.146	3.21	0.001	0.1393	0.0541	0.2245
Medication	1.1519	0.7597	-0.3408	2.645	1.52	0.130	0.0662	-0.0196	0.1521
Autism	-3.2791	1.0427	-5.3280	-1.230	-3.14	0.002	-0.1335	-0.2170	-0.0501
